# Microfluidic Capture-Enrichment of individualized microbes from urban wastewater reveals a hidden reservoir of potential pre-emergent pathogens

**DOI:** 10.64898/2026.09.21.753334

**Published:** 2026-09-23

**Authors:** Siyi Zhou, Seokju Seo, Kathryn Rhatigan, Marga Lee, Alice Liu, Alyse Tran, Fernanda Vaca, Lois Armendariz, Todd Treangen, Natalia V. Kirienko, Lauren B. Stadler, Yousif Shamoo

## Abstract

Current environmental biosurveillance infrastructure excels at tracking known biological threats but is limited in its ability to proactively identify potential pre-emergent pathogens (PEPs). To address this gap, we developed a microfluidics-based Capture-Enrichment pipeline that isolates individual environmental cells via microdroplet encapsulation, preserving community biodiversity while enabling iterative selection against human sera. When applied to urban wastewater, this approach successfully recovered pathogens, commensals, and PEPs that fell below the detection limits of conventional amplicon sequencing. Genomic analysis revealed that wastewater-derived ESKAPE pathogens cluster closely with clinical isolates, showing that the pipeline captures clinically relevant threats. Furthermore, isolated PEPs exhibited known and emerging phenotypes associated with early-stage pathogenesis and antimicrobial resistance. These findings demonstrate that targeting PEPs can fundamentally expand the scope and quality of biosurveillance by providing a scalable strategy to identify, characterize, and close important gaps in our understanding of emerging microbial pathogens.

## Full Text Availability

The license terms selected by the author(s) for this preprint version do not permit archiving in PMC. The full text is available from the preprint server.

